# Walking pace, handgrip strength, age, APOE genotypes, and new-onset dementia: the UK Biobank prospective cohort study

**DOI:** 10.1186/s13195-022-01158-6

**Published:** 2023-01-09

**Authors:** Panpan He, Chun Zhou, Ziliang Ye, Mengyi Liu, Yuanyuan Zhang, Qimeng Wu, Yanjun Zhang, Sisi Yang, Gan Xiaoqin, Xianhui Qin

**Affiliations:** 1 Division of Nephrology, Nanfang Hospital, Southern Medical University, Guangzhou, 510515 China; 2 National Clinical Research Center for Kidney Disease, Guangzhou, 510515 China; 3 State Key Laboratory of Organ Failure Research, Guangzhou, 510515 China; 4 Guangdong Provincial Institute of Nephrology, Guangdong Provincial Key Laboratory of Renal Failure Research, Guangdong Provincial Clinical Research Center for Kidney Disease, Guangzhou Regenerative Medicine and Health Guangdong Laboratory, Guangdong Provincial Clinical Research Center for Kidney Disease, Guangzhou, 510515 China

**Keywords:** Walking pace, Handgrip strength, Dementia, APOE ε4 dosage, Age

## Abstract

**Background:**

The independent and additive associations of walking pace and grip strength on dementia risk and the potential modifying effects of age, APOE phenotypes, and other dementia risk factors on the walking pace and dementia relationships demand further clarification. We aimed to investigate the independent and additive relationships of walking pace and handgrip strength on the risk of new-onset dementia and examine the potentially modifying effects of age, APOE phenotypes, lifestyle factors, and family history of dementia in the relationships.

**Methods:**

A total of 495,700 participants from the UK Biobank, who were free of dementia at baseline, were included in this study. Walking pace was self-defined as slow, average, or brisk. Handgrip strength was assessed by dynamometer and was divided into sex-specific quartiles. The APOE genotypes were determined by a combination variant of rs429358 and rs7412. Other dementia risk factors, including education, physical activity, hypertension, depression, diabetes, and family history of dementia, were also collected. The primary outcome was new-onset all-cause dementia.

**Results:**

Over a median follow-up duration of 12.0 years, 3986 (0.8%) participants developed new-onset all-cause dementia. Compared with those with slow walking pace, participants with average (HR, 0.61; 95%CI: 0.55–0.68) or brisk (HR, 0.59; 95%CI: 0.52–0.67) walking pace had a significantly lower risk of new-onset all-cause dementia. Moreover, compared with those with both slow walking pace and lower handgrip strength (the first quartile), the lowest risk of new-onset all-cause dementia was observed in participants with both average or brisk walking pace and higher handgrip strength (the 2–4 quartiles) (HR, 0.45; 95%CI: 0.40–0.52). Notably, the negative relationship between walking pace and the risk of new-onset all-cause dementia was significantly reduced as APOE ε4 dosage increased (APOE ε4 dosages = 0 or 1: brisk *vs.* slow: HR, 0.55; 95%CI: 0.48–0.63; *vs.* APOE ε4 dosages = 2: brisk *vs.* slow: HR, 1.14; 95%CI: 0.77–1.68; *P* for interaction = 0.001) or age increased (< 58 [median]: brisk *vs.* slow: HR, 0.27; 95%CI: 0.18–0.41; *vs.* ≥ 58 years: brisk *vs.* slow: HR, 0.55; 95%CI: 0.48–0.63; *P* for interaction = 0.007).

**Conclusions:**

Walking pace was inversely associated with new-onset dementia in the general population, especially in younger participants and those with lower APOE ε4 dosage. Participants with both faster walking pace and higher handgrip strength had the lowest risk of dementia, suggesting that maintaining both high handgrip strength and fast walking pace may be a more comprehensive strategy for preventing dementia risk.

**Supplementary Information:**

The online version contains supplementary material available at 10.1186/s13195-022-01158-6.

## Introduction

Dementia is a major public health concern, posing a substantial burden on patients, their carers, and national healthcare systems [1, 2]. Around 50 million people worldwide suffer from dementia, and this number is projected to increase to 152 million by 2050 [1]. There is currently no complete cure for dementia, so identifying potential risk factors may reveal opportunities for primary prevention. One area of interest is whether the physical function is related to dementia onset, as both of them involve the central nervous system and share some common age-related mechanisms [3, 4].

Walking pace has been recognized as a reliable, valid, and sensitive measure to screen older adults for subclinical pathogenetic processes that may impair overall health and function [5]. Several studies have shown that a brisk walking pace was associated with a lower risk of dementia [3, 5–14], while some studies have reported no significant association [15, 16]. Another measure of physical function is grip strength as it measures muscular strength and biological validity, and poorer grip strength has been reported to be associated with mortality, and other negative health outcomes [17]. Recently, some studies [14, 17–19] reported that lower grip strength was associated with a higher risk of all-cause dementia. However, from a physiological point of view, walking pace and grip strength may reflect different underlying physiological processes. Grip strength is more likely a measure of contraction strength whereas walking pace integrates strength with other processes, such as balance and coordination. As such, the combination of both may result in a stronger association with health outcomes than individually [20]. Previous studies have evaluated the combination of walking pace and grip strength on cardiovascular disease (CVD) [21, 22] and type 2 diabetes [20] and suggested that the combination of walking pace and grip strength was more strongly associated with health outcomes than in isolation. In addition, previous studies on the associations of walking pace and handgrip strength on cognitive decline [23, 24] have reported that when grip strength and walking pace were included in a regression model simultaneously, only walking pace was associated with cognitive decline. Meanwhile, none of the studies [23, 24] have examined the combined association of walking pace and grip strength on cognitive decline. More importantly, to the best of our knowledge, the combined association of walking pace and grip strength with the risk of dementia has not previously been investigated.

Furthermore, age; modifiable risk factors like obesity, physical activity, hypertension, depression, and diabetes; family history of dementia; and genetic factors like the carriage of the apolipoprotein E (APOE) ε4 allele have been associated with an elevated risk of overall dementia or Alzheimer’s disease (AD) [25, 26]. These factors, especially age and APOE, have also been associated with walking speed and handgrip [27, 28]. Therefore, they may represent important confounders or modifying factors of the relationships between walking pace or grip strength and new-onset dementia. It is of great importance to clinical and public health relevance to examine whether the association with dementia varies across those potential effect modifiers.

In this study, we took advantage of the large sample size of UK Biobank to (1) evaluate the individual and joint associations of walking pace and handgrip strength with the risk of new-onset dementia, and the major subtypes of dementia (AD and vascular dementia), and (2) to further examine the possible modifying effects of age, APOE ε4 dosage, family history of dementia, and dementia modifiable risk factors including obesity, physical activity, hypertension, depression, and diabetes on the walking pace and dementia associations.

## Methods

### Population and study design

The UK Biobank is a population-based cohort of more than 500,000 participants who attended 1 of 22 assessment centers across the UK between 2006 and 2010. At enrollment, participants completed a touch-screen questionnaire, had physical measurements taken, and provided biological samples, as described in detail elsewhere [29, 30].

The current analyses were restricted to participants who had self-reported measures of walking pace and grip strength measures and without self-reported or prevalent dementia at baseline. A total of 495,700 participants were included in the final analysis (eFigure 1).

This study is based on data from the UK Biobank study that received approval from the National Information Governance Board for Health and Social Care and the National Health Service North West Multicentre Research Ethics Committee. All participants gave written informed consent before enrollment in the study, which was conducted in accord with the principles of the Declaration of Helsinki.

### Exposure and covariates

The walking pace was self-reported via a touchscreen-based questionnaire by answering the question “How would you describe your usual walking pace? (i) Slow pace, (ii) Steady/average pace, and (iii) Brisk pace”. Handgrip strength was measured by using a Jamar J00105 hydraulic hand dynamometer while sitting. Isometric grip force was assessed from a single 3-s maximal grip effort of the right- and left-side arms with participants seated upright with their elbow by their side and flexed at 90° so that their forearm was facing forward and resting on an armrest. Both left and right hands strengths were measured. The mean of the right- and left-side values, expressed as kg, was used in the analysis. The measured handgrip strength was further divided into sex-specific quartiles as follows: Q1 (F: < 19.5 kg; M: < 34.0 kg), Q2 (F: 19.5 to < 23.0 kg; M: 34.0 to < 39.5 kg), Q3 (F: 23.0 to < 27.5 kg; M: 39.5 to < 45.0 kg), Q4 (F: ≥ 27.5 kg; M: ≥ 45.0 kg).

Blood collection sampling procedures for the study have previously been described and validated [30]. Biochemical assays were performed at a dedicated central laboratory. Multiple immunoassays and clinical chemistry analyzers were used to measure the biochemistry markers, details of which are provided in online companion documents (https://biobank.ndph.ox.ac.uk/ukb/ukb/docs/serum_biochemistry.pdf). Of which, C-reactive protein (CRP) levels were measured by immuno-turbidimetric method on a Beckman Coulter AU5800. Albumin levels were measured by colorimetric method on a Beckman Coulter AU5800. Body mass index (BMI) was calculated as weight (kg) by height squared (m^2^). Area-based socioeconomic status was derived from the postal code of residence by using the Townsend deprivation score. Smoking (never, former, current) and drinking (never or special occasions, 1–3 times/month, 1–2 times/week, 3–4 times/week, daily or almost daily) status, education levels (college or university degree, A levels/AS levels or equivalent, O levels/General Certificate of Secondary Education [GCSEs] or equivalent, Certificate of Secondary Education [CSEs] or equivalent, National Vocational Qualification [NVQ] or Higher National Diploma [NHD] or Higher National Certificates [HNC] or equivalent, other professional qualifications), and depression (yes or no) were self-reported at baseline. Family history of dementia (yes or no) was collected by answering the question “Has/did your mother/ father/ any of your brothers or sisters ever suffer from dementia?”. High education levels include college or university degrees and NVQ or NHD or HNC or equivalent. Optimal physical activity was defined as more than 4 days of vigorous/moderate physical activity in a typical week [31]. A healthy diet score [32, 33] was calculated based on the adequate intake of the following diet factors: increased consumption of fruits (≥ 3 servings/day), vegetables (≥ 3 servings/day), whole grains (≥ 3 servings/day), (shell)fish (≥ 2 servings/week), dairy products (≥ 2 servings/day), and vegetable oils (≥ 2 servings/day), and reduced or no consumption of refined grains (≤ 2 servings/day), processed meats (≤ 1 serving/week), unprocessed meats (≤ 2 servings/week), and sugar-sweetened beverages (do not drink). Each point was given for each favorable diet factor, and the healthy diet score ranged from 0 to 10. Seated blood pressure was measured twice manually (manual sphygmometer) or automatically (Omron HEM-7015IT digital blood pressure monitor), and the mean value of the two measurements was used to minimize measurement error. Hypertension was defined as systolic blood pressure (SBP) ≥ 140 mmHg, diastolic blood pressure (DBP) ≥ 90 mmHg, self-reported antihypertensive treatment or self-reported hypertension history, or International Classification of Diseases (ICD)-9 (401) or ICD-10 (I10). Prevalent diabetes (yes or no) at baseline was identified through multiple procedures considering the type of diabetes and sources of the diagnosis [34]. CVD (yes or no) at baseline was identified as a composite of preexisting coronary heart disease, myocardial infarction, ischemic heart disease, stroke, heart failure, and atrial fibrillation.

### Genetic risks of dementia

The APOE genotypes were determined by a combination variant of rs429358 and rs7412. Based on the number of APOE Ɛ4 alleles, participants were divided into high-risk group (APOE ε4 dosage = 2, ε4/ε4), normal-risk group (APOE ε4 dosage = 1, ε3/ε4), and low-risk group (APOE ε4 dosage = 0, ε2/ε2, ε2/ε3, ε3/ε3) in this analysis [26, 35].

Dementia genetic risk scores (without the AOPE genotype) were calculated by 25 single nucleotide polymorphisms (SNPs), which passed quality control, based on the previous study [36]. A weighted method was used to calculate the PRS [37]; higher scores indicated a higher genetic predisposition to AD and cognitive disorder. Further detailed information on genotyping, imputation, and quality control in the UK Biobank study has been described previously [38].

### Study outcomes

The primary outcome was new-onset all-cause dementia. The secondary outcomes included AD and vascular dementia. Diagnoses were recorded using the International Classification of Diseases (ICD9 and ICD10) coding system (eTable 1). The accuracy of dementia ascertainment has been validated previously [39].

Each participant’s person-years were calculated from the date of attending the assessment center to the date reported for diagnosis of new-onset dementia events, death, loss to follow-up, or end of the follow-up, whichever occurred first.

### Statistical analysis

Baseline characteristics were presented as mean (SD) for continuous variables or proportions for categorical variables by walking pace and sex-specific quartiles of handgrip strength. Differences in characteristics were compared using ANOVA tests or chi-square tests accordingly.

Basic demographic variables and variables known as traditional or suspected risk factors for dementia were selected as covariates. The relations of walking pace, handgrip strength with all-cause dementia, and the major subtypes of dementia (AD and vascular dementia) were estimated using Cox proportional hazards models (hazards ratio [HR] and 95% confidence interval [CI]) without (the crude model) and with adjustments for a series of covariates, including age, sex, ethnicities, Townsend deprivation score, BMI, smoking and alcohol drinking status, education levels, physical activity, healthy diet scores, CRP, albumin, CVD, hypertension, depression, diabetes, and family history of dementia in model 1. Moreover, APOE ε4 dosage and dementia genetic risk scores were further adjusted in model 2 and model 3, respectively. In model 4, walking pace and handgrip strength were further mutually adjusted.

We combined walking pace and handgrip strength into a category variable: group 1, slow walking pace and grip strength in the first quartile; group 2, slow walking pace and grip strength in the 2–4 quartiles; group 3, average or brisk walking pace and grip strength in the first quartile; group 4, average or brisk walking pace and grip strength in the 2–4 quartiles. Similar Cox proportional hazards models were used to examine the association of combined walking speed and handgrip strength with risk of all-cause dementia with group 1 as reference.

To evaluate the interactions between walking pace and age, APOE ε4 dosage, handgrip strength, or other confounding factors (BMI, education, physical activity, hypertension, depression, diabetes, and family history of dementia), multiplicative interactions were assessed by adding an interaction term to the Cox proportional hazards model.

A two-tailed *P* < 0.05 was considered to be statistically significant in all analyses. R software (version 4.1.3, http://www.R-project.org) was used for all statistical analyses.

## Results

### Baseline characteristics of participants

A total of 495,700 participants were included in the current study. The average age of the study population was 56.5 (SD, 8.1) years. 225,861 (45.6%) of the participants were male. 40,464 (8.2%), 261,751 (52.8%), and 193,485 (39.0%) participants reported slow, average, and brisk walking pace, respectively.

The baseline characteristics of study participants are presented by walking pace in Table 1. Participants who reported slow walking pace were older, more likely to be female, non-white, and smokers; had lower physical activity and education levels; less likely to drink and to have healthy diet scores, more likely to have CVD, hypertension, depression, and diabetes, and had higher deprivation index, BMI, CRP, APOE ε4 dosage, and lower albumin levels (all *P* values < 0.001).

**Table 1:** Population characteristics by walking pace categories

Characteristics	Walking pace			*P* value
	Slow	Average	Brisk	
*N*	40,464	261,751	193,485	
Age, year	58.8 (7.5)	57.1 (8.0)	55.2 (8.1)	< 0.001
Female, *n* (%)	22,378 (55.3)	143,128 (54.7)	104,333 (53.9)	< 0.001
White, *n* (%)	36,154 (89.9)	245,483 (94.1)	186,472 (96.7)	< 0.001
Deprivation index	0.0 (3.5)	− 1.3 (3.1)	− 1.6 (2.9)	< 0.001
Higher education, *n* (%)	9814 (25.0)	88,644 (34.5)	88,644 (34.5)	< 0.001
BMI, kg/m^2^	31.2 (6.5)	28.0 (4.7)	25.8 (3.8)	< 0.001
Optimal physical activity, *n* (%)	10,523 (30.6)	100,287 (41.2)	83,025 (44.4)	< 0.001
Healthy diet score	2.9 (1.4)	3.0 (1.4)	3.2 (1.4)	< 0.001
Smoking, *n* (%)				< 0.001
Never	17,807 (44.4)	140,676 (54.0)	112,149 (58.1)	
Former	15,272 (38.0)	91,877 (35.2)	64,081 (33.2)	
Current	7069 (17.6)	28,158 (10.8)	16,772 (8.7)	
Alcohol drinking, *n* (%)				< 0.001
Never or special occasions	15,393 (38.1)	53,066 (20.3)	27,706 (14.3)	
1–3 times/month	4690 (11.6)	30,588 (11.7)	20,006 (10.3)	
1–2 times/week	8900 (22.1)	69,318 (26.5)	49,920 (25.8)	
3–4 times/week	5660 (14.0)	57,622 (22.0)	51,402 (26.6)	
Daily or almost daily	5713 (14.2)	50,916 (19.5)	44,351 (22.9)	
Prevalent health conditions, *n* (%)				
CVD	9187 (22.8)	20,887 (8.0)	8793 (4.5)	< 0.001
Hypertension	29,114 (73.0)	153,871 (59.3)	92,856 (48.3)	< 0.001
Depression	4604 (11.4)	14,269 (5.5)	8771 (4.5)	< 0.001
Diabetes	6154 (15.2)	14,549 (5.6)	4963 (2.6)	< 0.001
Dementia family history	4670 (11.5)	30,531 (11.7)	22,668 (11.7)	0.594
CRP, mg/L	4.7 (6.5)	2.7 (4.3)	1.9 (3.5)	< 0.001
Albumin, g/L	44.5 (2.8)	45.1 (2.6)	45.5 (2.6)	< 0.001
APOE ε4 dosage				< 0.001
0	28,129 (74.0)	182,341 (73.6)	134,373 (73.1)	
1	9045 (23.8)	59,474 (24.0)	44,861 (24.4)	
2	817 (2.2)	6045 (2.4)	4591 (2.5)	

*Abbreviations: BMI* body mass index, *CVD* cardiovascular disease, *CRP* C-reactive protein. Variables are presented as mean (SD) or *n* (%)

Moreover, participants in lower quartiles of grip strength were younger; less likely to be white and current smokers; more likely to be alcohol drinkers; more likely to have hypertension, depression, diabetes, CVD, and dementia family history; had higher deprivation index, BMI, Cystatin C, and CRP levels and lower education, physical activity, and albumin levels (all *P* values < 0.001) (eTable 2).

### Associations of walking pace, handgrip strength, and combined walking pace and handgrip strength with new-onset dementia

During a median follow-up of 12.0 years (interquartile range, 11.2–12.7 years), a total of 3986 (0.8%) new-onset cases of dementia were documented, including 2638 cases of AD and 1404 cases of vascular dementia.

In the crude model, compared to slow walking pace, the HRs (95%CI) were 0.41 (0.37–0.44) and 0.28 (0.26–0.31) for average and brisk walking pace (*P* for trend < 0.001), respectively. After multivariate adjustments (model 1), the inverse association was attenuated but remained significant. Compared to those with slow walking pace, participants with average (HR, 0.61; 95%CI: 0.55–0.68) or brisk (HR, 0.59; 95%CI: 0.52–0.67) walking pace had a significantly lower risk of new-onset all-cause dementia (*P* for trend < 0.001). Similarly, compared to those with slow walking pace, participants with average or brisk walking pace had a significantly lower risk of new-onset AD (average *vs.* slow walking pace: HR, 0.64; 95%CI: 0.55–0.73; brisk *vs.* slow walking pace: HR, 0.64; 95%CI: 0.55–0.75; *P* for trend < 0.001) and vascular dementia (average *vs.* slow walking pace: HR, 0.54; 95%CI: 0.45–0.64; brisk *vs.* slow walking pace: HR, 0.47; 95%CI: 0.38–0.58; *P* for trend < 0.001) in model 1 (Table 2). There was no obvious change in the strength of the relationship between walking pace and new-onset dementia after further adjustments for APOE ε4 dosage in model 2 (average *vs.* slow walking pace: HR, 0.60; 95%CI: 0.54–0.67; brisk *vs.* slow walking pace: HR, 0.59; 95%CI: 0.52–0.67; *P* for trend < 0.001) and dementia genetic risk scores in model 3 (average *vs.* slow walking pace: HR, 0.61; 95%CI: 0.54–0.68; brisk *vs.* slow walking pace: HR, 0.59; 95%CI: 0.52–0.67; *P* for trend < 0.001) (Table 2).

**Table 2:** The association of walking pace with new-onset dementia and its major subtypes

	Walking pace			*P* for trend
	Slow *N* = 40,464	Average *N* = 261,751	Brisk *N* = 193,485	
**All-cause dementia**				
Events (%)	733 (1.8)	2093 (0.8)	1090 (0.6)	
Crude model	Ref	0.41 (0.37, 0.44)	0.28 (0.26, 0.31)	< 0.001
Model 1	Ref	0.61 (0.55, 0.68)	0.59 (0.52, 0.67)	< 0.001
Model 2	Ref	0.60 (0.54, 0.67)	0.59 (0.52, 0.67)	< 0.001
Model 3	Ref	0.61 (0.54, 0.68)	0.59 (0.52, 0.67)	< 0.001
Model 4	Ref	0.65 (0.58, 0.72)	0.64 (0.57, 0.73)	< 0.001
**Alzheimer’s disease**				
Events (%)	412 (1.0)	1424 (0.5)	802 (0.4)	
Crude model	Ref	0.49 (0.44, 0.55)	0.37 (0.33, 0.42)	< 0.001
Model 1	Ref	0.64 (0.55, 0.73)	0.64 (0.55, 0.75)	< 0.001
Model 2	Ref	0.63 (0.54, 0.73)	0.64 (0.55, 0.76)	0.001
Model 3	Ref	0.63 (0.55, 0.73)	0.64 (0.54, 0.75)	< 0.001
Model 4	Ref	0.67 (0.58, 0.78)	0.70 (0.60, 0.82)	0.005
**Vascular dementia**				
Events (%)	369 (0.9)	732 (0.3)	303 (0.2)	
Crude model	Ref	0.28 (0.25, 0.32)	0.16 (0.13, 0.18)	< 0.001
Model 1	Ref	0.54 (0.45, 0.64)	0.47 (0.38, 0.58)	< 0.001
Model 2	Ref	0.53 (0.44, 0.62)	0.47 (0.38, 0.58)	< 0.001
Model 3	Ref	0.53 (0.45, 0.63)	0.47 (0.38, 0.58)	< 0.001
Model 4	Ref	0.56 (0.48, 0.67)	0.51 (0.41, 0.62)	< 0.001

Model 1 adjusted for age, sex, ethnicities, BMI, socioeconomic deprivation, smoking and alcohol drinking status, education levels, physical activity, healthy diet scores, C-reactive protein (CRP), albumin, cardiovascular disease (CVD), hypertension, depression, diabetes, and dementia family history

Model 2 adjusted for covariates in model 1 plus APOE ε4 dosage

Model 3 adjusted for covariates in model 1 plus dementia genetic risk scores

Model 4 adjusted for covariates in model 1 plus grip strength

Moreover, when handgrip strength was assessed as sex-specific quartiles, in the crude model, compared to the first quartile, the HRs (95%CI) were 0.59 (0.55–0.64), 0.36 (0.33–0.40), and 0.20 (0.18–0.22) for the second, third, and fourth quartile (*P* for trend < 0.001), respectively. After multivariate adjustments (model 1), the inverse association was attenuated but remained significant. Compared to those in the first quartile, significantly lower risks of new-onset dementia were found in participants in the second (HR, 0.77; 95%CI: 0.71–0.85), third (HR, 0.67; 95%CI: 0.60–0.74), and fourth quartiles (HR, 0.58; 95%CI: 0.51–0.66), respectively, after multivariate adjustments (model 1) (eTable 3). As such, participants in the first quartile of handgrip strength were defined as having lower handgrip strength. Similar results were found when the walking pace and handgrip strength were further mutually adjusted in model 4 (Table 2, eTable S3).

**Fig. 1:**
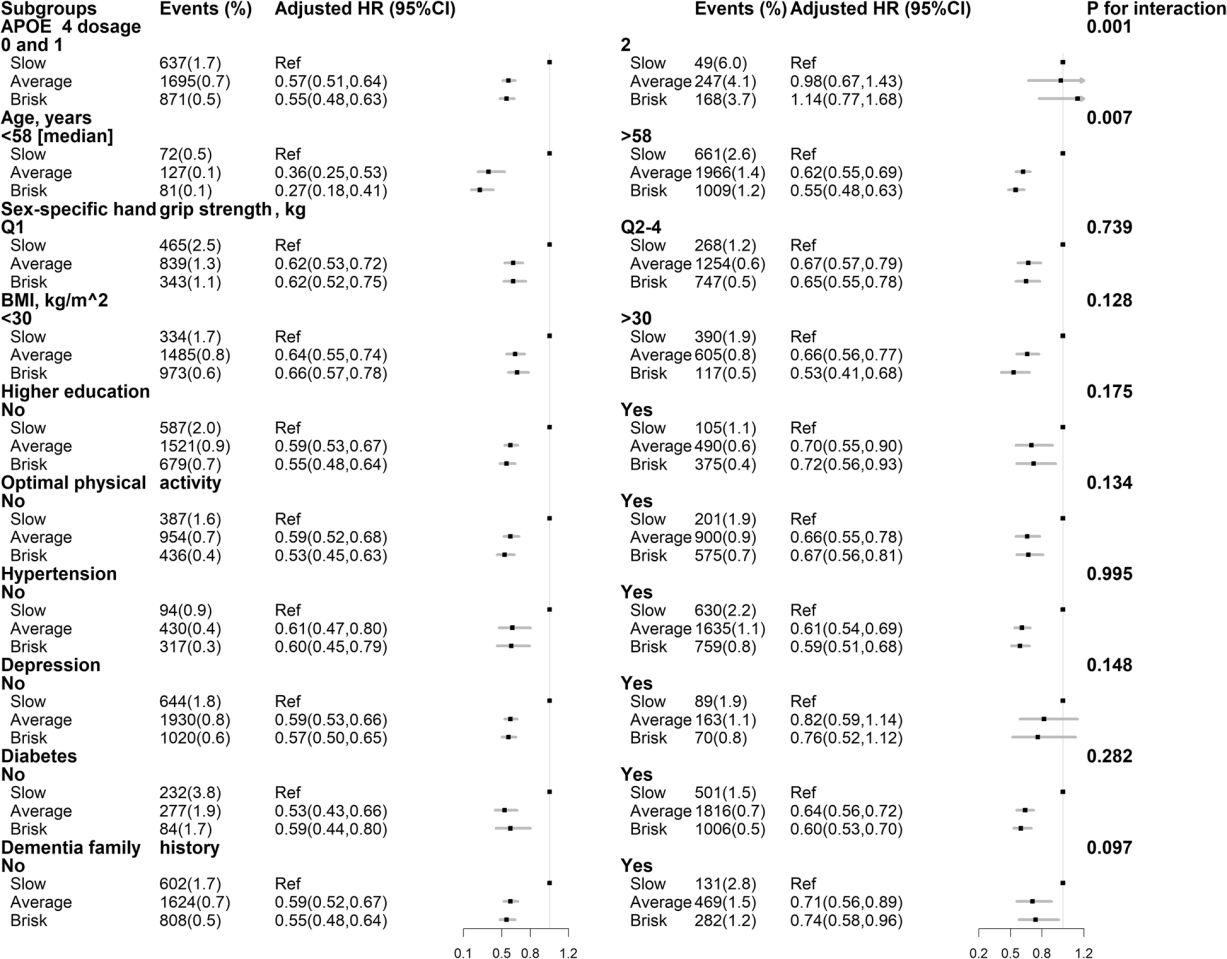
Stratified analyses by potential effect modifiers for the association between walking pace and new-onset dementia. Adjusted for age, sex, ethnicities, BMI, socioeconomic deprivation, smoking and alcohol drinking status, education level, physical activity, healthy diet score, C-reactive protein (CRP), albumin, cardiovascular disease (CVD), hypertension, depression, diabetes, and dementia family history

Although there was no statistically significant interaction between walking pace and handgrip strength on new-onset dementia (*P* for interaction = 0.739) (Fig. 1), compared to those with both lower handgrip strength (the first quartile) and slow walking pace, the lowest risk of new-onset dementia was observed in participants with both higher handgrip strength (the 2–4 quartiles) and average or brisk walking pace (adjusted HR, 0.45; 95%CI: 0.40–0.52) (model 2) (Table 3).

**Table 3:** The joint association of walking pace (slow *vs.* average or brisk) and handgrip strength (the first quartile *vs.* the 2–4 quartiles) with new-onset dementia

Group^a^	*N*	Events (%)	Crude model		Adjusted model	
			HR (95%CI)	*P* value	HR (95%CI)	*P* value
1	18,722	465 (2.5)	Ref		Ref	
2	21,742	268 (1.2)	0.46 (0.39, 0.53)	< 0.001	0.68 (0.57, 0.82)	< 0.001
3	94,171	1182 (1.3)	0.47 (0.42, 0.52)	< 0.001	0.62 (0.54, 0.72)	< 0.001
4	361,065	2001 (0.6)	0.19 (0.17, 0.21)	< 0.001	0.45 (0.40, 0.52)	< 0.001

Adjusted for age, sex, ethnicities, BMI, socioeconomic deprivation, smoking and alcohol drinking status, education levels, physical activity, healthy diet scores, C-reactive protein (CRP), albumin, cardiovascular disease (CVD), hypertension, depression, diabetes, and family history of dementia

^a^Group 1, slow walking pace and grip strength in the first quartile;

Group 2, slow walking pace and grip strength in the 2–4 quartiles;

Group 3, average or brisk walking pace and grip strength in the first quartile;

Group 4, average or brisk walking pace and grip strength in the 2–4 quartiles

### Stratified analyses

Stratified analyses were performed to assess the relationship between walking pace and new-onset dementia in various subgroups.

Walking pace was differently related to risks of new-onset dementia among participants with different APOE ε4 dosages (*P* for interaction = 0.001). In the low or normal risk groups (APOE ε4 dosage = 0 or 1), walking pace was significantly associated with a lower risk of new-onset dementia (brisk vs. slow: HR, 0.55; 95%CI: 0.48–0.63), while in the high-risk group (APOE ε4 dosage = 2), walking pace was not significantly associated with the risk of new-onset dementia (brisk vs. slow: HR, 1.14; 95%CI: 0.77–1.68) after multivariate adjustments (model 1) (Fig. 1). Moreover, a stronger inverse association between walking pace and new-onset dementia was found in younger participants (< 58 [median]: brisk *vs.* slow: adjusted HR, 0.27; 95%CI: 0.18–0.41; *vs.* ≥ 58 years: brisk *vs.* slow: adjusted HR, 0.55; 95%CI: 0.48–0.63; *P* for interaction = 0.007) (Fig. 1).

None of the other variables, including BMI, education, physical activity, hypertension, depression, and diabetes, significantly modified the association between walking pace and all-cause dementia (all *P* for interactions > 0.05) (Fig. 1). The walking pace and all-cause dementia association was consistent in those subgroups.

## Discussion

In this large population-based prospective cohort study, we found that self-perceived walking pace was inversely associated with the risk of new-onset dementia. The lowest risk of new-onset dementia was observed in participants with both faster walking pace and higher handgrip strength. More importantly, the inverse association between walking pace and new-onset dementia was significantly attenuated as APOE ε4 dosage or age increased.

Some previous studies with relatively small sample sizes have shown that brisk walking pace was related to lower risk dementia [3, 5, 7–12], whereas other studies conducted in the elderly population [15, 16] found that gait speed was not associated with dementia. Recently, a large cohort study also demonstrated that slow walking pace was associated with higher risks of all dementia types [14]. However, to date, the combined association of walking pace and grip strength with the risk of dementia, as well as the modifying effects of age, APOE phenotypes, and other important dementia risk factors, including obesity, physical activity, hypertension, depression, and diabetes, on the relationship between walking pace and new-onset dementia, have not been comprehensively investigated. Our current study, with a magnitude larger sample size and more extensive adjustments for confounding factors, including lifestyle, clinical measures, and genetic characteristics, addresses the knowledge gap in this field in a timely manner.

Our study showed that in the crude models, there was a significant inverse association between walking pace and the risk of dementia. Moreover, in the joint analysis of walking pace and handgrip strength, those with both slow walking pace and low handgrip strength had the highest dementia risk. Of note, these associations were obviously attenuated after adjustments for multiple potential confounders such as physical activity, healthy diet scores, hypertension, depression, diabetes, and family history of dementia, but remained significant, suggesting that the associations were partly independent of these important traditional risk factors. These results have biological plausibility. The widespread network of brain areas that control walking involves regions responsible for attentional, executive, and visuospatial functions, as well as regions required for performing and controlling motor tasks, such as the cerebellum, basal ganglia, and motor cortex [40]. Thus, there is an overlap between areas that control walking and areas that control cognitive function, which may explain the relationship between dementia-related pathology and gait dysfunction.

Our findings further expand the results of previously published studies on the walking pace and dementia associations [14] by demonstrating that the APOE ε4 dosage significantly modified the association between walking pace and new-onset dementia. APOE-ε4 allele, especially participants with two alleles, has been associated with several biochemical processes implicated in the etiology of AD, including amyloid deposits, neurofibrillary tangles formation, neuronal cell death, oxidative stress, neuroinflammation, synaptic alterations, and cholinergic signaling dysfunction [41]. More importantly, the detrimental effects of APOE ε4 on the neuronal system are irreversible [42]. Therefore, the already increased dementia risk in APOE ε4 carriers may attenuate the inverse relations between faster walking pace and the risk of dementia.

In addition, our results show that the inverse association between walking pace and new-onset dementia was significantly stronger in younger participants. Age is undoubtedly an important risk factor for dementia. We hypothesized that age may explain part of the association between walking pace and dementia, and therefore the relation of walking pace with new-onset dementia was significantly attenuated among older participants who were already at higher risk for age-related dementia. Further mechanistic studies are required to unravel the pathways involved in those associations.

Several potential limitations of the current study need to be addressed. First, the walking pace was self-reported, so there might be bias in its measurement. However, the self-reported walking pace item used in UK Biobank has been shown to be associated with cardiorespiratory fitness [43], and self-reported walking pace has more generally been observed to be strongly associated with objectively assessed walking pace [44]. The use of self-reported walking pace is a potential strength as it is a simple-to-measure risk factor that can easily be incorporated into future research and clinical care pathways at low cost. Second, though a broad range of potential confounding was included in the adjustments, possible effects from other unknown or unmeasured covariates could not be excluded. Third, participants in the UK Biobank are primarily of White British origin. Consequently, findings might not be generalizable to other ethnicities or populations. Fourth, UK Biobank participants are not representative of the general population and hence cannot be used to provide representative disease prevalence and incidence rates. Thus, further confirmation of the reported findings in future studies is necessary.

## Conclusions

In conclusion, our findings indicate that a faster self-perceived walking pace was inversely associated with the risk of new-onset dementia. The lowest risk of new-onset dementia was observed in participants with both average or brisk walking pace and higher handgrip strength. More importantly, the inverse association between walking pace and new-onset dementia was significantly attenuated as APOE ε4 dosage or age increased. Our findings highlight the importance of assessing combined measures of physical function, genetic profile, and age to improve the stratification of individuals at risk of dementia.

## Supplementary Information

**Additional file 1: eFigure 1.** Flow chart of study participants. **eTable 1.** Codes used in the UK Biobank to identify dementia cases. **eTable 2.** Population characteristics by sex-specific handgrip strength quartiles. **eTable 3.** The association between handgrip strength and new-onset dementia*.

## Data Availability

Researchers can apply to use the UK Biobank resource and access the data used. No additional data are available. The analytic code will be made available from the corresponding authors on request.
