# Correction: Walking pace, handgrip strength, age, APOE genotypes, and new-onset dementia: the UK Biobank prospective cohort study

**DOI:** 10.1186/s13195-023-01223-8

**Published:** 2023-04-04

**Authors:** Panpan He, Chun Zhou, Ziliang Ye, Mengyi Liu, Yuanyuan Zhang, Qimeng Wu, Yanjun Zhang, Sisi Yang, Gan Xiaoqin, Xianhui Qin

**Affiliations:** 1 Division of Nephrology, Nanfang Hospital, Southern Medical University, Guangzhou, 510515 China; 2 National Clinical Research Center for Kidney Disease, Guangzhou, 510515 China; 3 State Key Laboratory of Organ Failure Research, Guangzhou, 510515 China; 4 Guangdong Provincial Institute of Nephrology, Guangdong Provincial Key Laboratory of Renal Failure Research, Guangdong Provincial Clinical Research Center for Kidney Disease, Guangzhou Regenerative Medicine and Health Guangdong Laboratory, Guangdong Provincial Clinical Research Center for Kidney Disease, Guangzhou, 510515 China

**Correction: Alz Res Ther 15, 9 (2023)**

10.1186/s13195-022-01158-6

Following publication of the original article [1], there were errors in the supplementary material of eTable 1. The correct description should be as attached. This error did not affect the data analyses and results as presented in the paper.

**eTable 1. Codes used in the UK Biobank to identify dementia cases**

**Table:** 

	ICD-9	ICD-10
All-cause dementia	331.0, 290.4, 331.1	F00, F00.0, F00.1, F00.2, F00.9, G30, G30.0, G30.1, G30.8, G30.9, F01, F01.0, F01.1, F01.2, F01.3, F01.8, F01.9, I67.3, F02.0, G31.0
Alzheimer’s Disease	331.0	F00, F00.0, F00.1, F00.2, F00.9, G30, G30.0, G30.1, G30.8, G30.9
Vascular Dementia	290.4	F01, F01.0, F01.1, F01.2, F01.3, F01.8, F01.9, I67.3

The original article [1] has been updated.
