# Supplementary material for: Walking pace, handgrip strength, age, APOE genotypes, and new-onset dementia: the UK Biobank prospective cohort study
Source: Alzheimers Res Ther. 2023 Jan 9;15:9. doi: 10.1186/s13195-022-01158-6 (PMC9827642; doi:10.1186/s13195-022-01158-6)
Supplement: Supplementary file 1 — Additional file 1: eFigure 1. Flow chart of study participants. eTable 1. Codes used in the UK Biobank to identify dementia cases. eTable 2. Population characteristics by sex-specific handgrip strength quartiles. eTable 3. The association between handgrip strength and new-onset dementia*. [file 13195_2022_1158_MOESM1_ESM.docx]

Original dataset

N= 502,414

Participants enrolled

N= 501,405

Excluded: self-reported or prevalent dementia at baseline N= 1009

Final analysis

N= 495,700

Excluded:

Missing baseline handgrip strength measurement, N= 1518;

Missing baseline walking pace information, N= 4187

**eFigure 1. Flow chart of study participants**

**eTable 1. Codes used in the UK Biobank to identify dementia cases**

|  | **ICD-9** | **ICD-10** |
| --- | --- | --- |
| All-cause dementia | 331.0, 290.4, 331.1, 290.2, 290.3, 291.2, 294.1, 331.2, 331.5 | F00, F00.0, F00.1, F00.2, F00.9, G30, G30.0, G30.1, G30.8, G30.9, F01, F01.0, F01.1, F01.2, F01.3, F01.8, F01.9, I67.3, F02.0, G31.0, A81.0, F02, F02.1, F02.2, F02.3, F02.4, F02.8, F03, F05.1, F10.6, G31.1, G31.8 |
| Alzheimer’s Disease | 331.0 | F00, F00.0, F00.1, F00.2, F00.9, G30, G30.0, G30.1, G30.8, G30.9 |
| Vascular Dementia | 290.4 | F01, F01.0, F01.1, F01.2, F01.3, F01.8, F01.9, I67.3 |

**eTable 2.** **Population characteristics by sex-specific handgrip strength quartiles**

| Characteristics | Sex-specific handgrip strength, kg | | | | *P* value |
| --- | --- | --- | --- | --- | --- |
|  | Q1 | Q2 | Q3 | Q4 |  |
| N | 112893 | 122186 | 129188 | 131433 |  |
| Age, year | 59.4 (7.4) | 58.0 (7.7) | 56.1 (7.9) | 53.0 (7.9) | < 0.001 |
| Male, n (%) | 55744 (49.4) | 56706 (46.4) | 51771 (40.1) | 61640 (46.9) | < 0.001 |
| White, n (%) | 103805 (92.4) | 115797 (95.1) | 123336 (95.8) | 125171 (95.5) | < 0.001 |
| Deprivation index | -0.9 (3.3) | -1.3 (3.1) | -1.5 (3.0) | -1.5 (3.0) | < 0.001 |
| Higher education, n (%) | 35847 (32.5) | 43700 (36.4) | 50079 (39.4) | 55735 (43.0) | < 0.001 |
| BMI, kg/m^2 | 27.8 (5.0) | 27.3 (4.7) | 27.2 (4.7) | 27.4 (4.7) | < 0.001 |
| Optimal physical activity, n (%) | 42177 (41.3) | 47599 (41.7) | 50872 (41.6) | 53187 (42.1) | 0.002 |
| Healthy diet scores | 2.4 (0.9) | 2.4 (0.9) | 2.4 (0.9) | 2.4 (0.9) | < 0.001 |
| Smoking, n (%) |  |  |  |  | < 0.001 |
| Never | 60133 (53.6) | 66331 (54.5) | 71237 (55.3) | 72931 (55.6) |  |
| Former | 39950 (35.6) | 43004 (35.3) | 44533 (34.6) | 43743 (33.4) |  |
| Current | 12196 (10.9) | 12383 (10.2) | 13014 (10.1) | 14406 (11.0) |  |
| Alcohol drinking, n (%) | |  |  |  | < 0.001 |
| Daily or almost daily | 29173 (25.9) | 24112 (19.8) | 23092 (17.9) | 19788 (15.1) |  |
| 3-4 times/week | 12154 (10.8) | 13424 (11.0) | 14613 (11.3) | 15093 (11.5) |  |
| 1-2 times/week | 27753 (24.6) | 31439 (25.8) | 33731 (26.1) | 35215 (26.8) |  |
| 1-3 times/month | 22440 (19.9) | 27997 (22.9) | 30829 (23.9) | 33418 (25.4) |  |
| Never or special occasions | 21185 (18.8) | 25105 (20.6) | 26848 (20.8) | 27842 (21.2) |  |
| Prevalent health conditions, n (%) | |  |  |  |  |
| CVD | 15614 (13.9) | 11165 (9.2) | 8807 (6.8) | 6670 (5.1) | < 0.001 |
| Hypertension | 69531 (62.2) | 70257 (57.9) | 69457 (54.2) | 66596 (51.1) | < 0.001 |
| Depression | 7657 (6.8) | 6827 (5.6) | 6981 (5.4) | 6179 (4.7) | < 0.001 |
| Diabetes | 9722 (8.6) | 6290 (5.1) | 5040 (3.9) | 3828 (2.9) | < 0.001 |
| Dementia family history | 14591 (12.9) | 15221 (12.5) | 15265 (11.8) | 12792 (9.7) | < 0.001 |
| Laboratory results |  |  |  |  |  |
| Cystatin C, mg/L | 1.0 (0.2) | 0.9 (0.2) | 0.9 (0.2) | 0.9 (0.1) | < 0.001 |
| CRP, mg/L | 3.2 (5.1) | 2.6 (4.3) | 2.4 (4.1) | 2.2 (3.7) | < 0.001 |
| Albumin, g/L | 44.8 (2.7) | 45.1 (2.6) | 45.3 (2.6) | 45.6 (2.6) | < 0.001 |
| APOE ε4 dosage |  |  |  |  | < 0.001 |
| 0 | 78698 (73.9) | 85187 (73.6) | 89649 (73.2) | 91309 (73.0) |  |
| 1 | 25324 (23.8) | 27690 (23.9) | 29659 (24.2) | 30707 (24.6) |  |
| 2 | 2506 (2.4) | 2845 (2.5) | 3090 (2.5) | 3012 (2.4) |  |

**Abbreviations:** body mass index (BMI), cardiovascular disease (CVD), C-reactive protein (CRP)

Variables are presented as Mean (SD) or n (%).

**eTable 3. The association between handgrip strength and new-onset dementia**^*^

|  | Sex-specific handgrip strength, kg | | | | *P* for trend |
| --- | --- | --- | --- | --- | --- |
|  | Q1, N= 112893 | Q2, N= 122186 | Q3, N= 129188 | Q4, N= 131433 |  |
| Events (%) | 1647(1.5) | 1107(0.9) | 737(0.6) | 425(0.3) |  |
| Crude Model | Ref | 0.59(0.55,0.64) | 0.36(0.33,0.40) | 0.20(0.18,0.22) | <0.001 |
| Model 1 | Ref | 0.77(0.71,0.85) | 0.67(0.60,0.74) | 0.58(0.51,0.66) | <0.001 |
| Model 2 | Ref | 0.78(0.71,0.85) | 0.66(0.60,0.74) | 0.59(0.51,0.67) | <0.001 |
| Model 3 | Ref | 0.77(0.71,0.85) | 0.67(0.60,0.74) | 0.58(0.51,0.66) | <0.001 |
| Model 4 | Ref | 0.80(0.73,0.87) | 0.69(0.62,0.77) | 0.61(0.53,0.69) | <0.001 |

^*^ Model 1 adjusted for age, sex, race, BMI, socioeconomic deprivation, smoking and alcohol drinking status, education levels, physical activity, healthy diet scores, C-reactive protein (CRP), albumin, cardiovascular disease (CVD), hypertension, depression, diabetes and family history of dementia;

Model 2 adjusted for covariates in Model 1 plus APOE ε4 dosage;

Model 3 adjusted for covariates in Model 1 plus dementia genetic risk scores**;**

Model 4 adjusted for covariates in Model 1 plus walking pace.
